# The burden of disease and economic growth: The nonlinear effect of population age structure

**DOI:** 10.1016/j.heliyon.2024.e30119

**Published:** 2024-04-20

**Authors:** Flora Aninditya, Omas Bulan Samosir, Hera Susanti, Mahjus Ekananda

**Affiliations:** a Vocational Education Program, Universitas Indonesia, Depok, 16424, Indonesia; b Lembaga Demografi, Faculty of Economics and Business, Universitas Indonesia, Depok, 16424, Indonesia; c Department of Economics, Faculty of Economics and Business, Universitas Indonesia, Depok, 16424, Indonesia

**Keywords:** Global burden of disease, Economic growth, Population age structure, Threshold regression, Nonlinear effect

## Abstract

This study aims to detangle the impact of health on economic growth as empirical evidence shows a mixture of findings. We use data on the Burden of Disease (BoD) from the Institute of Health Metric Evaluation (IHME) to measure health capital and the economic data of 87 countries from 1990 to 2018. Using panel threshold regression, this study shows that the old dependency ratio is a good measure of the threshold variable, which divides the country groups into four. The BoD, whether it comes from communicable diseases (CD), non-communicable diseases (NCD), or injuries, has a negative impact on economic growth. However, the negative relation is somewhat diminished as the population gets older, demonstrating that the BoD's impact on economic growth is less pronounced for the older population than the younger population.

## 1 Introduction

Although the positive impact of health on economic growth is widely recognized [[1], [2], [3]] recent empirical evidence shows a mixed and inconclusive relation between the two [[4], [5], [6], [7]].

This study aims to fill the literature gap by examining the relationship between the two, considering two emerging issues: (1) the changing profile of population health and (2) the changing population age structure.

In the last decades, along with economic development, there have been dramatic changes in the global health profile due to demographic changes and the global epidemiological transition [8]. In previous studies, life expectancy was mainly used as the proxy of the population's health [[9], [10], [11]]. However, it may not be adequate to capture the quality of the living. This study proposes using the disease burden as a more appropriate proxy to explain the population health profile.

Unlike previous studies that acknowledge the different impacts of health on economic growth due to different levels of a country's development [12] and longevity [13], this study emphasizes how different population age structures might explain the different impacts of diseases on economic growth. Specifically, we use the old age dependency ratio as a threshold variable that differentiates the populations.

## 2 Literature review

Past studies have proposed a single notion primarily about the relationship between health and economic growth. However, recent studies demonstrate that the relationship between the two is suggested to be positive, negative, inconclusive, and non-linear. A study examines the impact of life expectancy among 17 advanced countries for 143 years, using the dynamic panel analysis, and finds that life expectancy affects economic growth positively [11]. Another study among OECD countries also find that life expectancy among OCED countries during the period 1940–1980 positively and significantly affects economic growth [9]. Similar approach was used to examine the impact of longevity on economic growth on 200 countries during 1960–2000 period, using a data set of a five-year average from the annual data observation. Confirming previous studies, they find that longevity, proxied by life expectancy, positively affects economic growth [10].

The idea of a positive relationship between health and economic growth is challenged by some influential studies that show a mixed and inconclusive relationship between the two. A study in 2007 claim that there is no indication that as life expectancy increases, per capita income also increases [4]. Increases in life expectancy have a negligible or negative effect on per capita income. A follow up study was conducted using the same dataset along by adding initial life expectancy and the lagged effect of life expectancy, employing non-linear GMM into the model [5]. Even after incorporating suggested approaches, there is no proof of the positive impact of life expectancy on GDP per capita. A simulation for different countries and years and found that an increase in life expectancy lead to a positive effect in economic growth in the long run but less than previous estimates [6].

A negative impact of health on economic growth are also proposed by some other studies [5,14], even after incorporating initial life expectancy into the model, assuming the initial condition among countries will affect the economic growth [2,4]. It seems that life expectancy has a negative impact on economic growth, and that countries with high life expectancy growth tend to have lower per capita GDP growth [14].

Most earlier studies are also built on the assumption of the linear relationship among health and economic growth. Nonetheless, some other studies propose the non-linear relationship among the two. Life expectancy and GDP per capita actually has U-shaped relationship and that the effect of health on wealth or income depends on development level [13] and longevity [12]. With a life expectancy of less than 43 years in 1940, life expectancy has been found to have a negative impact on income per capita [12].

The result differences found in existing studies imply that the health and economic growth nexus may vary considerable in magnitude. The variability results of how health affects economic growth might be attributed to differences in the context of the studies, such as the health proxy used, the distinction between less developed and developed countries, the different stages of demographic transition, and of the gender perspectives. In light of this, scholars introduce the using of threshold regression approach to capture the effect of health on economic growth in different sub samples to demonstrate the existence of non-linear health-economic growth effects.

A study that employs panel data from 1996 to 2014 among Sub-Saharan countries, uses the ratio of public health expenditure as the threshold variable. The effect of life expectancy is found to be higher when accompanied by a minimum of 3.5 % of the ratio of public health expenditure to GDP [15]. Still using the threshold approach, another study found that life expectancy is significant when it exceeds a threshold and insignificant when the life expectancy is below a threshold level [16]. Meanwhile, another study found that when life expectancy increases, it will lead to an increase in economic growth as well, particularly when the level of life expectancy is low [17]. Another study [18] also suggested that life expectancy has a significant effect when controlled with spending on health and education. A threshold effect could also explains human capital and economic growth relation [19]. The level of human capital may vary depending on the development of the economy, whether it is underdeveloped or developed.

To select the relevant variable used for the threshold variable, it is important to consider that changes in age structure play important roles in its effect on income per capita. The demographic transition in Asia has been acknowledged as an important driver for economic growth, with different implications in East Asia and Southeast and South Asia [20]. Similar notion is applied in African countries where countries with high level of fertility are expected to experience higher income per capita as the level of fertility began to decline [21]. Another study suggests that an increase of one percentage point of the share of the working age population may improve 1.5 percentage points of GDP per capita [22]. Thus, this study proposes the use of old-dependency ratio, a measure of population age structure, as the threshold variable.

Old-age dependency ratio (*odr*) is defined by comparing those who are 65 or older with those between the ages 15 and 64. Between 1990 and 2019, the global *odr* keeps increasing. In 2019, for every 100 people aged 20 to 64, there were 16 older people aged 65 or more. Although the *odr* is built on the assumption that the elderly people are economically dependent upon the younger population, this measure still can provide information on how health impacts differently across the populations.

## 3 Method

Generally, the inclusion of health to economic growth model can be started by assuming an aggregate production function as follows:

(1) $$Y_{t}=K_{t}^{\alpha }E_{t}^{\beta }H_{t}^{\rho }{\left[A_{t}L_{t}\right]}^{\mu }$$

Where μ is defined as 1- α-β-ρ, and that α,β,ρ > 0, and 0 < μ < 1. Here, Y_t_ is aggregate output; K_t_ is the physical capital; E_t_ and H_t_ represent the aggregate human capital in the form of education and health, respectively. Equation 2.1 then can be expressed in terms of effective labor unit by dividing it by AL.

(2) $$y_{t}=k_{t}^{\alpha }\;e_{t}^{\beta }h_{t}^{\rho }$$

Where $y_{t}=\frac{Y}{AL}$, $k_{t}=\frac{K}{AL}$, $e_{t}=\frac{E}{AL}$ , $h_{t}=\frac{H}{AL}$.

The equation then has been extended by involving numbers of dimensions or factors affecting economic growth (e.g openness, inflation, investment) [23]. For empirical purposes, the analysis in this study is built upon panel data analysis. However, basic panel regression model would not be able to expose the true nature of the health and economic growth as the age structure changes. Considering this, this study then adopts threshold regression model [24]. Threshold regression is an extended version of linear regression that allows coefficients to differ across groups. This method is developed for non-dynamic panels with individual specific fixed effects.

The basic form of threshold regression is as follow:

$$y_{it}=\alpha_{i}+\beta_{1}^{\prime }x_{it}+\varepsilon_{it}\;if\;q_{it}\leq c$$

(3) $$y_{it}=\alpha_{i}+\beta_{2}^{\prime }x_{it}+\varepsilon_{it}\;if\;q_{it}>\;c$$

Here c is the threshold parameter, q is the predictor with threshold effect, x denotes additional predictors. The observations are divided into some “regimes”, whether the threshold variable is higher or lower than the threshold c. A given cross section unit i, may have different slope parameters, split by c. The slopes $\beta_{1}^{\prime }$ and $\beta_{2}^{\prime }$ reflects the different slopes of the regime. Threshold variable can be determined on the basis of an exogenous variable. For this study, proposed variable to be used as the threshold is the old dependency ratio that represents the aging structure of population. As the objective of the study is to capture the impact of health on economic growth considering the different of population age structure, the specification can be rewritten into three models, each model incorporates different type of the burden of diseases. Three models are used to represent each type of the burden of diseases, namely the communicable diseases (CD), non-communicable diseases (NCD) and injuries (*ldalycd, ldalyncd, ldalyinj*).

Model 1 Impact of burden of communicable diseases to economic growth.

(4) $${lgdppc}_{i,t}=\mu_{i}+{\gamma_{1,i}ldalycd}_{i,t}+{\gamma_{2}schooling}_{i,t}+{{\gamma_{3}invest}_{i,t}+{\gamma_{4}inflation}_{i,t}+{\gamma_{5}open}_{i,t}+{+u}_{i,t}+\varepsilon }_{i,t\;}if\;odr\leq c$$

(5) $${lgdppc}_{i,t}=\mu_{i}+{\gamma_{1,i}ldalycd}_{i,t}+{\gamma_{2}schooling}_{i,t}+{{\gamma_{3}invest}_{i,t}+{\gamma_{4}inflation}_{i,t}+{\gamma_{5}open}_{i,t}+{+u}_{i,t}+\varepsilon }_{i,t\;}if\;{odr}_{i,t}>\;c$$

Model 2 Impact of burden of injuries to economic growth.

(6) $${lgdppc}_{i,t}=\delta_{i}+{\rho_{1,i}ldalyinj}_{i,t}+{\rho_{2}schooling}_{i,t}+{{\rho_{3}invest}_{i,t}+{\rho_{4}inflation}_{i,t}+{\rho_{5}open}_{i,t}+{+u}_{i,t}+\varepsilon }_{i,t\;}if\;{odr}_{i,t}\leq c$$

(7) $${lgdppc}_{i,t}=\delta_{i}+{\rho_{1,i}ldalyinj}_{i,t}+{\rho_{2}schooling}_{i,t}+{{\rho_{3}invest}_{i,t}+{\rho_{4}inflation}_{i,t}+{\rho_{5}open}_{i,t}+{+u}_{i,t}+\varepsilon }_{i,t\;}if\;{odr}_{i,t}>\;c$$

Model 3 Impact of burden of non-communicable diseases to economic growth.

(8) $${lgdppc}_{i,t}=\sigma_{i}+{\theta_{1,i}ldalyncd}_{i,t}+{\theta_{2}schooling}_{i,t}+{{\theta_{3}invest}_{i,t}+{\theta_{4}inflation}_{i,t}+{\theta_{5}open}_{i,t}+{+u}_{i,t}+\varepsilon }_{i,t\;}\;if\;{odr}_{i,t}\leq c$$

(9) $${lgdppc}_{i,t}=\sigma_{i}+{\theta_{1,i}ldalyncd}_{i,t}+{\theta_{2}schooling}_{i,t}+{{\theta_{3}invest}_{i,t}+{\theta_{4}inflation}_{i,t}+{\theta_{5}open}_{i,t}+{+u}_{i,t}+\varepsilon }_{i,t\;}\;if\;{odr}_{i,t}>\;c$$

Two main data sources used in this study are data on the economy and health status at the country level for 87 countries. They are five years averaged data over the 1990–2018 period among 87 countries. Data is averaged over 1990–1994, 1995–1999, 2000–2004, 2005–2009, 2010–2014, 2015–2018, so that provides a maximum of 6 observations per country. Economic growth is expressed in terms of GDP per capita at purchasing power parity and is expressed in the natural logarithm. Data on economy is drawn from World Development Indicators. In addition, mean years of schooling is included as control variables.

Data on BoD is represented by Disability-Adjusted Life Years (DALYs-hereafter) from International Health Metric Evaluation (IHME). Specifically, we use the age-standardized DALY rates. A DALY represents a one-year loss of an individual's fully healthy years due to premature death and/or being in poor health due to illness or disability. In this study, the mortality (Years Life Lost/YLL) and morbidity (Years Living with Disabilty/YLD) components were not analyzed separately. Instead, we separate the analysis into three categories of DALYs: communicable diseases, injuries, and non-communicable diseases.

## 4 Results

Table 1, Table 2 provide some elementary descriptive statistics and coefficient correlation between variables. Furthermore, to work with threshold panel data, some diagnostic tests must be performed: (1) Hausman Test, to find if the fixed-effects or random-effects model is preferred, and (2) Non-linearity Test and Threshold Effect Test, to examine if the model has non-linearity. The results show that the p-value for the three Hausman tests is less than 0.05 for all models, meaning that the null hypothesis is rejected and that the preferred method for further analysis is fixed effects, rather than random effects.

**Table 1:** Descriptive statistics.

	Mean	Min.	Max.	Std. Dev.	Obs.
lgdppc	9.35	6.67	11.29	1.16	522
open	79.11	14.38	407.12	55.44	522
inflation	14.57	−4.24	1667.15	104.22	522
investgdp	21.99	4.50	46.22	6.13	522
schooling	7.54	0.76	14.10	3.08	522
odr	12.55	2.84	44.48	8.05	522
ldalycd	8.71	6.52	11.33	1.29	522
ldalyncd	9.98	9.43	10.52	0.19	522
ldalyinj	8.16	7.23	10.35	0.43	522

**Table 2:** Correlation matrix.

	lgdppc	open	inflation	investgdp	schooling	dro	ldalycd	ldalyncd	ldalyinj
**lgdppc**	1								
**open**	0.3913	1							
**inflation**	−0.045	−0.0909	1						
**investgdp**	0.2014	0.158	−0.0596	1					
**schooling**	0.8541	0.2986	−0.0639	0.1457	1				
**odr**	0.6916	0.0965	−0.051	−0.0053	0.7103	1			
**ldalycd**	−0.8843	−0.3034	0.0575	−0.2262	−0.728	−0.7762	1		
**ldalyncd**	−0.5935	−0.2592	0.0586	−0.2208	−0.615	−0.6257	0.7006	1	
**ldalyinj**	−0.5812	−0.3552	0.1005	−0.1768	−0.5736	−0.5624	0.634	0.604	1

As shown in the threshold-effect test table (Table 3), *Single* corresponds to H_0_ (linear model) and Ha (single threshold model), *Double* corresponds to H_0_ (single-threshold model) and Ha (double threshold model), and so forth. For all three models, the double-threshold models are accepted with a probability value of 0.0200, 0.0333, and 0.0200. Therefore, the linear model is rejected and the models fit a double threshold model. The test results also confirm the non-linearity correlation between the burden of diseases (*ldalycd*, *ldalyncd*, and *ldalyinj*) on economic growth, considering the different levels of *odr* across countries. Having a double-threshold for all models means that in each and every model, there are three intervals produced. The first is below the first threshold, the second is between the first and second threshold value, and the third interval is above the second threshold value.

**Table 3:** Summary of the threshold effect test.

Threshold test	*P*-value	F-value	Critical value			BS times
			1 %	5 %	10 %	
Model 1 (*ldalycd)*						
Single***	0.00	81.21	38.23	33.34	27.22	300
Double**	0.02	34.41	40.92	29.90	25.12	300
Triple	0.52	26.51	77.26	62.74	53.07	300
Model 2*(ldalyinj)*						
Single***	0.00	99.57	42.54	31.7	26.17	300
Double**	0.02	39.13	42.76	29.51	25.63	300
Triple	0.48	32.3	80.69	65.88	58.68	300
Model 3*(ldalyncd)*						
Single***	0.003	97.46	39.18	30.40	26.46	300
Double**	0.033	34.3	39.53	32.15	25.89	300
Triple	0.41	36.56	88.27	67.98	59.76	300

Note: (1) Model 1 explores the population aging threshold effect between the burden of communicable diseases and economic growth, while Model 2 explores the population aging threshold effect between the burden of non-communicable from injuries and economic growth, and Model 3 displays the population aging threshold between the burden of non-communicable diseases and economic growth (2) ***, ** and * meant that the values are significant at 1 %, 5 % and 10 %, respectively. (3) The *P*-value, the F-value, and the critical value are the results of applying the bootstrap to repeat the sampling 300 times.

The specific threshold results are presented in Table 4 along with their confidence intervals of 95 %. Variable *ldalycd* from Model 1 and variable *ldalyinj* from Model 2 has the same estimated thresholds: 10.27 and 13.28. However, they possess different lower and upper values. Model 3's estimated threshold variables are 7.64 and 10.27. Double threshold estimators allow each of the models to have three intervals. For Models 1 and 2, the intervals of the population aging are (1) less than 10.27, (2) between 10.27 and 13.28, and (3) above 13.28. Meanwhile, for Model 3, the intervals are (1) less than 7.64, (2) between 7.64 and 10.27, and (3) beyond 10.27.

**Table 4:** Estimated threshold variables.

Threshold variables		Estimated threshold	95 % Confidence Interval	
			lower	upper
ldalycd	d1	10.27	10.20	10.32
	d2	13.28	12.98	14.26
ldalyinj	d1	10.27	9.928	10.32
	d2	13.28	13.04	14.26
ldalyncd	d1	7.64	7.52	10.32
	d2	10.27	10.21	14.26

Table 5, Table 6, Table 7 display the results analysis of each burden of disease to economic growth. In these models, two thresholds are used to divide population aging into three intervals. In the early stages of population aging, the burden of communicable diseases in these countries increases. When the *odr* is between the thresholds of 10.27 and 13.28, the coefficient is −0.24. This interval indicates a negative correlation, and it is less sensitive than the first interval. When the level of population aging crosses the second threshold of 13.28 the coefficient is −0.20. The negative impact of CD on economic growth is somehow weakened, which means that in the later stage of the aging population, the economic growth decreases compared to the previous ones. Same threshold values from Model 1 are also found in Model 2 that links the burden of diseases from injuries to economic growth. Where the *odr* is below 10.27, the coefficient of *ldalyinj* negative and significant at the 1 % significance level with a value of −0.15. When the *odr* is between the thresholds of 10.27 and 13.28, the coefficient is −0.13. When the *odr* is above 13.28, the coefficient is −0.09.

**Table 5:** Results of the fixed effect and threshold model for Model 1.

	Fixed Effect		Threshold Model					
			(odr <10.2659)		(10.2659 <odr <13.2801)		(odr >13.2801)	
**ldalycd**	−0.3352	***	−0.2602	***	−0.2392	***	−0.2030	***
schooling	0.0583	***	0.0748					***
investgdp	0.0075	***	0.0073					***
infla	0.0000		0.0000					
open	0.0005		0.0011					***
dro	0.0151	***						
								
constant	11.44		10.66					
observations	522		522					
R-squared within	0.64		0.69					
F-test	63.31		73.53					
# of groups	87		87					

***, ** and * indicate significance at of 1 %, 5 % and 10 %, respectively.

**Table 6:** Results of the fixed effect and threshold model for Model 2.

	Fixed Effect		Threshold Model					
			(d < 10.2659)		(10.2659<d < 13.2801)		(d > 13.2801)	
**ldalyinj**	−0.2073	***	−0.1525	***	−0.1268	***	−0.0892	***
schooling	0.1047	***	0.1071					***
investgdp	0.0113	***	0.0102					***
infla	0.0000		0.0000					
open	0.0003		0.0010					***
dro	0.0130	***						
constant	9.82		9.30					
observations	522		522					
R-squared within	0.60		0.67					
F-test	79.29		95.86					
# of groups	87		87					

***, ** and * indicate significance at of 1 %, 5 % and 10 %, respectively.

**Table 7:** Results of the fixed effect and threshold model for Model 3.

	Fixed Effect		Threshold Model					
			(d < 7.6424)		(7.6424<d < 10.2659)		(d > 10.2659)	
**ldalyncd**	−0.5814	***	−0.5153	***	−0.5011	***	−0.4760	***
schooling	0.1021	***	0.0921					***
investgdp	0.0115	***	0.0092					***
infla	0.0000		0.0001					
open	0.0000		0.0008					***
dro	0.0110	***						
constant	13.99		13.34					
observations	522		522					
R-squared within	0.64		0.67					
F-test	79.47		97.95					
# of groups	87		87					

***, ** and * indicate significance at of 1 %, 5 % and 10 %, respectively.

This interval indicates a negative correlation, and the latest is less sensitive than the former intervals. In other words, this shows how the negative correlation between the burden of diseases coming from injuries and economic growth is weakened, which means that in the later stage of an aging population, the elasticity coefficient of economic growth keeps decreasing compared to the previous ones. Model 3 shows the burden of non-communicable diseases to economic growth. In this mode, the threshold values are 7.64 and 10.27. When *odr* is less than the threshold value of 7.64, the coefficient is −0.52, which is significant at 1 %.

To sum up, the BoD, whether in the form of disease of CD, injuries or NCD, have a negative impact on economic growth along with the increase in population aging. However, the impact is weakening as the population gets older. In a younger population, the impact of the burden of communicable diseases (*ldalycd*) and injuries (*ldalyinj*) on economic growth is larger than in a relatively older population. In an older population, the impact is slightly reduced. Similar findings are drawn from the burden of non-communicable diseases (*ldalyncd*). In sum, as the population gets older, the impact of the BoD on economic growth is lesser than the younger population. Although all forms of the burden of diseases show a similar pattern toward economic growth, the impact of *ldalyncd*, shown by their coefficient is larger compared to the impact of *ldalycd* and *ldalyinj* (see Model 1 and Model 2). Except for inflation, the control variables, the openness, mean years of schooling, and the ratio of investment to GDP are all positive, and all have a positive impact on economic growth at the significance level of 1 %.

## 5 Discussion

To draw a possible explanation for these results, it is important to explore the profile of the countries and sample observations covered in the study. A combined pattern of observations from all models of the BoD shows that there are four patterns or groups identified (Table 8, Table 9). First, countries which all observations for Models 1, 2, and 3 falls under the third regimes (above the first threshold) across periods (27 countries). Second, countries whose all observations are always included in the first regimes (below the first threshold) are 23 countries. Third, countries where all observations for Model 1 and 2 are always under the first regime, while for Model 3, the observations lie across periods and intervals (15 countries). Lastly, countries whose observations are spread across regimes and periods for all Models (22 countries).

**Table 8:** Distribution of countries according to regime or threshold interval (A).

CD/Injuries	NCD		
	below 1st threshold (younger pop.)	between 1st and 2nd thresholds (in between)	beyond 2nd threshold (older pop.)
**below 1**st **threshold (younger pop.)**	23 countries	15 countries	
**between 1**st **and 2**nd **thresholds (in between)**			
**beyond 2**nd **threshold (older pop.)**			27 countries

**Table 9:** Distribution of countries according to regime or threshold interval (B).

CD/Injuries	NCD		
	below 1st threshold (younger pop.)	between 1st and 2nd thresholds (in between)	beyond 2nd threshold (older pop.)
**below 1**st **threshold (younger pop.)**	22 countries		
**between 1**st **and 2**nd **thresholds (in between)**			
**beyond 2**nd **threshold (older pop.)**			

By grouping countries according to the regimes or intervals they fall into, a deeper understanding can also be drawn. A combined pattern of observations from all models of the BoD shows that there are four patterns or groups identified. First, countries which all observations for Models 1, 2, and 3 falls under the third regimes (above the first threshold) across periods (27 countries). Countries included in this group are Japan, Australia, New Zealand, Argentina, Uruguay, Barbados, Bulgaria, Austria, Belgium, Cyprus, Denmark, Finland, France, Germany, Greece, Ireland, Italy, Luxembourg, Malta, Netherlands, Norway, Portugal, Spain, Switzerland, United Kingdom, and the United States. These countries maintain a relatively smaller impact from the all types of BoD on economic growth.

Second, countries whose all observations are always included in the first regimes (below the first threshold) are 23 countries. This group includes Brunei Darussalam, Pakistan, Honduras, Central African Republic, Burundi, Kenya, Tanzania, Botswana, Eswatini, Zimbabwe, Cameroon, Cote d’Ivoire, Gambia, Ghana, Mali, Mauritania, Niger, Senegal, Togo, Sudan, Bahrain, Jordan, and Saudi Arabia. These countries always have a relatively larger impact of all types of burden of diseases on economic growth.

Third, countries where all observations for Model 1 and 2 are always under the first regime, while for Model 3, the observations lie across periods and intervals (15 countries). Countries included in this group are Indonesia, Malaysia, Philippines, Bangladesh, India, Nepal, Fiji, Haiti, Guatemala, Paraguay, South Africa, Algeria, Iran Morocco, and Egypt. Having all observations for Model 1 and 2 under the first threshold means that these countries always experience a relatively high impact of the burden of communicable diseases and injuries. Meanwhile, for burden coming from noncommunicable diseases, they encounter higher impact of the burden of noncommunicable diseases for some observations and relatively lower impact for some other observations. Lastly, countries whose observations are spread across regimes and periods for all Models (22 countries). Countries involved in this group are China, Korea, Thailand, Vietnam, Singapore, Sri Lanka, Chile, Dominican Republic, Jamaica, Bolivia, Ecuador, Peru, Colombia, Costa Rica, El Salvador, Mexico, Panama, Brazil, Gabon, Mauritius, Tunisia, and Turkey.

In general, the burden of disease may affect economic growth through various pathways. First, it causes mortality and directly reduces the number of active workers. Second, it boosts morbidity and reduces the number of active labor by decreasing working hours and decreasing the number of people who can work. However, statistics show that the magnitude of the impact is different according to the age structure of the countries and the pattern of the regimes where the countries are grouped. The pattern may vary substantially under different settings. Many countries with sizeable old populations are high-income countries (HICs). On the other hand, many low- and middle-income countries (LMICs) are still relatively young. Countries with an older population and have a negative yet smaller impact on the burden of communicable diseases and injuries are predominantly countries from Western Europe and Australasia. In contrast, countries with a relatively younger population have a negative and relatively larger impact on the burden of communicable diseases and injuries and are dominated by countries from Africa, Latin America, Southeast Asia, and South Asia.

Further, from Table 8, Table 9, we identify four categories. Countries categorized into the first group are relatively older and experience lesser impact than countries in other groups. They experienced a relatively lower impact of the burden of diseases from CD, NCD, and injuries on economic growth. They had a dependency ratio of older people more than the second threshold during observation periods from 1990 to 2018. When the burden of diseases increases, whether it comes from any disease, economic growth only decreases slightly. These countries are regarded as high-income countries as well. For these HICs, population aging has taken different trends than other groups defined in this study. As the population gets older, both morbidity and mortality caused by communicable diseases increase [25,26]. Older people, even in HICs, are at risk of being exposed not only to non-communicable diseases and injuries but also to communicable diseases that have a negative impact on economic growth.

An aspect that differentiates HICs to other country groups, that may also explain the lesser impact of the BoD is the more advanced health care system in the HICs. Although experiencing an increasing burden of diseases, people living in developed or high-income countries may benefit from a more established health system and infrastructure. Over time, healthcare spending as a proportion of GDP also tends to rise. Yet, public spending is a necessary but not sufficient condition for producing effective healthcare results. United States, for example, scores considerably lower than any other country on the combined metrics of health care performance but plainly spends more on health as a proportion of GDP. Various systems also have varied consequences for access to critical care and disparities within specific countries. The United Kingdom, for example, has the lowest percentage of persons reporting cost of health care and income disparity [27]. Compared to those in LICs, poor older women living in rural areas with diseases bear a larger economic burden while handling the burden of the disease due to limited access and high treatment cost [3].

Countries categorized into the second group have a considerably bigger impact of the burden from communicable, injuries, and non-communicable diseases on economic growth and have a dependency ratio of older persons that is always smaller than the first threshold during the observation period from 1990 to 2018. In other words, because these nations are relatively younger, they always have a lower effect than those in other categories. When the burden of diseases grows, regardless of the type of disease, economic growth diminishes considerably large. These countries appear to be considered low-income as well.

Among the younger population, NCD such as diabetes, cancer, heart diseases increased significantly. The underlying factors or risk factors for the increasing NCD are linked to unhealthy behaviors such as unhealthy diets and intakes, alcohol consumption, and less physical activity. Socioeconomic and cultural factors condition some activities like alcohol consumption and smoking. As the economy is expected to benefit from the demographic dividend, pre-conditions such as healthy, educated, skilled, and productive young population are necessary. As the absolute number of the current young cohort is much larger than the old cohort that they will replace in the future, it is imperative to devote programs to curb the rising trend of non-communicable diseases. In terms of injuries, the impact of injuries on economic growth is exacerbated in LIC, especially with limited trauma treatment and rehabilitation facilities, as well as limited enabling and supportive infrastructure. In addition, as car ownership increases, road-related fatalities also increase [28].

A variety of issues also confront low-income countries. These countries bore heaviest burdens related to metabolic risk and cardiovascular diseases in particular [29]. The BoD is quite high and effective medical facilities are scarce, resulting in self-medication for the majority of ailments. Self-medication is commonly found in developing countries, where it becomes inferior good for high income households. However, under certain circumstances where medical care is not available or extremely expensive, people prefer self-medication solution. Self-medication has a number of potential drawbacks, such as inaccurate self-diagnosis and self-treatment, medicine misuse due to wrong dose or prolonged usage, and adverse effects.

The third group consists of countries whose observations always show a higher impact of CD and injuries, while the impact of NCD are spread into various regimes during the period of 1990–2018. Meanwhile, the fourth group consists of countries whose observations are spread across periods and intervals. Countries under the third and fourth groups are dominated by middle-income countries (MICs). Countries that belong to this group mostly come from MICs and undergo demographic change and health development. Common infectious diseases found in these countries are tuberculosis and malaria. For example, in China, the incidence of pneumonia and tuberculosis in older people is high and it is expected to increase among those who live in rural and men. With the intensification of infectious diseases, more studies on prevention and treatments will be beneficial [30]. Furthermore, in the MICs, health care has been transitioning toward an ideal and better system. However, there are major challenges in these countries, such as a lack of financial support for those who require health care because the health spending is dominated by out of pocket spending; a lack of demand for effective interventions; budget misallocation; weak management in health sector, and bureaucracy [31]. With sets of barriers to access the needed interventions and lack of health infrastructure, people experience difficulties in getting the treatments and prolonging their burden of diseases.

In addition to the challenges in health care, these countries are also characterized by an urbanization and rural-out migration that causes modernization and changing health related aspects. As the world is growing more urbanized gradually, whereas Asia and Africa are predicted to have the biggest increases in urban population over the next 30 years, global health and the increasing of CD are faced with a number of issues. New megacities may act as breeding grounds for new epidemics, and zoonotic diseases may spread more quickly and pose hazards on a global scale [32]. Due to societal changes, time constraints, and altered eating behaviors, NCD such diabetes, cardiovascular disease, and chronic obstructive pulmonary disease are also quickly rising. NCD exacerbate the burden of CD, which remain significant contributors to morbidity and mortality, due to the impact of expanding metropolitan or urban areas on the profile of CD. Cities, in particular, possess the capacity to transform into environments that foster outbreaks.

From four identified groups, Table 10 sums up the impact of BoD to economic growth and the proposed explanation that shape the context for each group. At least four aspects may explain the impact difference across these countries: healthcare, budget, health behavior, and the respected country development. These differences, nevertheless, will cause implications and suggest different policy responses across countries.

**Table 10:** Summary of the impact of BoD on economic growth by country groups.

	Older and High-income Countries	Younger and Low-income countries	Middle-income countries (a)	Middle-income countries (b)
Burden of Disease	Negative, relatively small	Negative, relatively high	Negative, relatively high for CD and moderate for NCD	Negative, moderate for CD and NCD
**General characteristics of each group**				
Healthcare	Well established	Early stage of development	Transitioning/developing	Transitioning/developing
Budget	High	Low	Budget Misallocation	Budget Governance
Health behaviour	Unhealthy lifestyle	Traditional healers, self-medication	Unhealthy lifestyle	Unhealthy lifestyle
Development		Motorization	Urbanization	Urbanization

## 6 Conclusion

This study provides evidence of a non-linear relationship between health and economic growth in 87 countries during the period 1990–2018. In particular, the empirical results confirm the potential threshold level of population age structure that could explain BoD differential effects on economic growth. The impact of all types of BoD is found larger under the high regime of the old dependency ratio.

We discovered that the predicted BoD has a negative and statistically significant effect on economic growth and we identified four stylized country groupings that show the different context of how BoD impacts the economic growth. The BoD affect economy differently across countries between relatively younger population structure and older population structure. Our analysis also shows that the negative impact of BoD to economic growth is somewhat diminished as the population gets older, demonstrating that the BoD's impact on economic growth is less pronounced for older population than the younger population. The smaller impact is partly due to the countries' economic capacity, which are often high-income countries.

Although the magnitude of BoD's impact varied among each group, all groups experienced the same negative impact. Thus, it is imperative for countries in every group to persistently work towards minimizing the negative effects of disease burden on economic development. For example, the ageing population is exposed not only to NCD due to unhealthy behavior in the past or due to degenerative issues, but also to CD as the BoD from immunological health problems increase. This emphasizes the need to introduce programs to ease the NCD impact and vaccination of older people to tackle the CD. This approach is particularly relevant for countries from the first group. Concerning the younger population or countries from group two, as the absolute number of the current young cohort is much larger than the old cohort that they will replace in the future, it is also imperative to devote programs to curb the rising trend of non-communicable diseases. Moreover, the need to curb the NCD should be acquired from early life. Among all countries from groups three and four, programs to increase access to health care and preventive programs are also essential.

With the frequency of CD rising, governments and stakeholders may want to implement policies that successfully reduce disease incidence and its implications. Several measures are feasible to minimize the prevalence of NCD. Various medical investments, for example, have been advocated because they are cost-effective and simple to implement within the limits of LMICs. These measures include raising alcohol and cigarette taxes and lowering salt consumption in food. However, it will be difficult to apply without a full evaluation of the economic advantages of these interventions, as well as the predicted returns on investment for various policy alternatives. This is also applicable to many proposed programs, not only in the health sector but also in other areas, such as infrastructure for ensuring road safety or when other health issues in NCD, CD, and injuries remain prominent.

A thorough analysis of the economic impact of NCD will thus provide evidence on which types of policies are viable and whether investments in health in general and chronic conditions, in particular, are likely to provide sufficient returns on investment to justify their implementation. Thus, the calculation of the economic burden of NCD is critical for offering cost-benefit interventions and providing potential policy initiatives based on their return on investment. A recent study in South Africa proposed a simulation of the potential economic loss that can be prevented from investment in type 2 diabetes intervention [33]. Similar attempts could be reproduced in various economic and population setting such as the four country categories identified in this study. There is also an urgent need to improve the treatment facilities to curb the diseases resulting from injuries. It may require more investment in health professionals, treatment and physical facilities, and medical supplies.

There are also policy instruments beyond the field of public health that must be enabled. It is worth emphasizing that population health is crucial as it contributes to the labor productivity. The BoD would affect the supply of labor, as people will work in less hours or work with less productivity. Investment in the health sector to improve the quality of human capital and productivity and to minimize the loss of quality labor is then imperative. Thus, it is essential to provide equal training opportunities for those with some types of difficulties or disabilities, encouraging employers to remove physical barriers to employment, and offering work flexibility for early return to work. These labor market policies can enable people with physical limitations or disabilities to remain productive and employed. Healthy older people can also contribute to economic growth by working directly or indirectly in the labor force or participating in community initiatives.

Several limitations and suggestions for future research are as follows. First, the DALY measures used in the study are not disaggregated by sex and diseases. Future research may need to consider the effect of the BoD that is disaggregated by population sex, as the population structure or profile does not only change by age, but also by sex profile. The use of certain groups of diseases can also be explored as different regions or countries may be highly exposed to certain diseases. Second, this study also employs single measures of DALYs and does not distinguish the measures of morbidity (proxied by YLD) and mortality (proxied by YLL). Further studies may incorporate these data and isolate the partial effect of YLD and YLL otowards economic growth. Third, this study assumes BoD as an exogenous variable. Further research may employ the BoD as an endogenous variable. The dynamic panel threshold approach might be used in future research to study the different impacts of illness burden on economic growth via interactions with another form of human capital, namely education. Fourth, further studies may also incorporate and link the BoD with relevant issues such as the utilization and coverage of health services and the risk factors that contribute to the increasing BoD (such as unhealthy diet and physical inactivity).

## Data availability statement

Data will be made available on request.

## Funding

This research was supported by the Ministry of Education, Culture, Research and Technology of the Republic of Indonesia (NKB-882/UN2.RST/HKP.05.00/2022).

## CRediT authorship contribution statement

**Flora Aninditya:** Writing – review & editing, Writing – original draft, Validation, Supervision, Software, Project administration, Methodology, Investigation, Data curation, Conceptualization, Formal analysis, Visualization. **Omas Bulan Samosir:** Writing – original draft, Supervision, Methodology, Formal analysis, Conceptualization. **Hera Susanti:** Writing – original draft, Supervision, Formal analysis, Conceptualization. **Mahjus Ekananda:** Writing – original draft, Supervision, Formal analysis, Conceptualization, Methodology.

## Declaration of competing interest

The authors declare that they have no known competing financial interests or personal relationships that could have appeared to influence the work reported in this paper.
